# Fasting blood glucose-to-glycated hemoglobin ratio and all-cause mortality among Chinese in-hospital patients with acute stroke: a 12-month follow-up study

**DOI:** 10.1186/s12877-022-03203-3

**Published:** 2022-06-20

**Authors:** Zhong-ming Cai, Man-man Zhang, Ren-qian Feng, Xu-dong Zhou, Hao-man Chen, Zhi-peng Liu, Yan-zhi Wu, Qun-li Lin, Sheng-lie Ye, Cheng-wei Liao, Xue-rong Huang, Le-qiu Sun, Bo Yang, Bei-lei Zhu

**Affiliations:** 1 Department of First Clinical Medical School, The First Affiliated Hospital of Wenzhou Medical University, Wenzhou, PR China; 2 Department of Neurology, The First Affiliated Hospital of Wenzhou Medical University, Wenzhou, PR China; 3 Department of Neurology, Pingyang People’s Hospital, Wenzhou, Zhejiang, PR China; 4 Department of Neurology, Yongjia People’s Hospital, Wenzhou, Zhejiang, PR China; 5 Department of Neurology, Ruian People’s Hospital, Wenzhou, Zhejiang, PR China; 6 Department of Neurology, Wenzhou Medical University Affiliated Yueqing Hospital, Wenzhou, Zhejiang, PR China; 7 School of Public Health and Management, Wenzhou Medical University, Wenzhou, Zhejiang, PR China

**Keywords:** Acute stroke, All-cause mortality, Stress hyperglycemia, Fasting blood glucose, Glycated hemoglobin

## Abstract

**Background:**

Stroke is a leading cause of death and functional impairment in older people. To assess the prospective association between fasting blood glucose-to-glycated hemoglobin ratio and all-cause mortality and poor prognosis in stroke patients.

**Methods:**

A total of 971 Chinese inpatients with acute stroke (mean age of 65.7) were consecutively enrolled in the prospective clinical study and followed up for 12 months after discharge. Stress hyperglycemia was measured using the ratio of fasting blood glucose (FBG, mmol/L)/glycated hemoglobin (HbA1c, %). The primary outcome was all-cause mortality, and secondary outcomes were poor prognosis defined as infectious complications, a National Institutes of Health Stroke Scale (NIHSS) score ≥ 6, a Barthel Index score ≤ 60, or a modified Rankin Scale (mRS) score of 3–6, presented as multivariate-adjusted odds ratios (ORs) with 95% confidence intervals (CIs) across the quartiles of the FBG/HbA1c ratio.

**Results:**

There were 35 (4.1%) all-cause deaths at 3 months and 85 (11.4%) at 12 months. The inpatients with the highest quartile of the FBG/HbA1c ratio had a higher risk of all-cause death at 3 months (adjusted OR: 5.16, 95% CI: 1.03–25.74) and at 12 months (adjusted OR: 2.59, 95% CI: 1.14–5.89)) and a higher risk of infectious complications (adjusted OR 2.37, 95% CI 1.27–4.43) and dysfunction (adjusted OR 1.79, 95% CI 1.06–3.01) during hospitalization than inpatients with the lowest quartile.

**Conclusions:**

Stress hyperglycemia, measured by the FBG/HbA1c ratio, was associated with an increased risk of adverse outcomes, including all-cause death, infectious complications, and dysfunction after stroke.

**Supplementary Information:**

The online version contains supplementary material available at 10.1186/s12877-022-03203-3.

## Background

Stress hyperglycemia, a relatively transient increase in blood glucose levels secondary to neurohormonal derangements and inflammation [1], is associated with increased morbidity and mortality in in-patients hospitalized for myocardial infarction [2], congestive cardiac failure [3], and other critical illnesses [4]. Stress hyperglycemia may occur in a high proportion of patients with stroke [5]. During period of acute stroke, an increase in blood glucose levels was detected induced by the release of stress hormones after the stress stimulating the hypothalamus–pituitary–adrenal axis and the sympathetic nervous system [6]. Previous studies have shown that stress hyperglycemia appears to be a marker of an increased risk of short-term mortality and poor functional outcomes after stroke [6–8]. However, these studies were performed in selected patient populations, focusing on patients with acute ischemic stroke [6, 9] or further extending to the large vessel occlusion subtype [7]. Additionally, the prognosis was measured only during the hospital stay [9] or at three months after discharge [6, 7]. Furthermore, although specific guidelines for the definition of stress hyperglycemia have not been developed, most previous studies have been based on absolute, rather than relative increases in fasting or randomized blood glucose levels [8, 9].

Due to successful standardization, the glycated hemoglobin (HbA1c) concentration has been proposed to be a valuable diagnostic tool for monitoring long-term glycemic control [10]. Additionally, transient changes in blood glucose levels generally do not affect HbA1c concentration [11]. Recent studies have shown that relative hyperglycemia, defined as the fasting blood glucose (FBG)/HbA1c ratio [12–14] or as admission glucose divided by the estimated average glucose level derived from HbA1c [15], might be a better predictor of outcomes of critical illness than absolute glucose indicators. Therefore, it can be hypothesized that the FBG-to-HbA1c ratio could reflect stress hyperglycemia considering the background glucose level prior to the stroke event [12–15].

Infectious complications, including pneumonia, urinary tract infection (UTI), gastrointestinal infection, infectious fever etc. are the third most common stroke complications [16], affecting a large number of stroke populations [17]. Infection has been shown to be an independent predictor of neurological functional deterioration [18] and stroke readmission rates [19], leading to a substantial economic burden upon stroke care [20]. Stress hyperglycemia results in impaired host defenses, increasing the risk for infection after stroke [21], which may contribute to adverse outcomes jointly in stroke patients.

We designed this study to prospectively explore the association between stress hyperglycemia, measured by the FBG/HbA1c ratio, and all-cause mortality and poor prognosis after acute stroke. Furthermore, we assessed whether infectious complications may play an important role in the pathway from stress hyperglycemia to mid- and long-term all-cause death.

## Methods

The patient data collection and assessment of outcomes were described as our previous studies [22, 23].

### Patients

Five major medical institutions in Wenzhou (the First Affiliated Hospital of Wenzhou Medical University, Yueqing People’s Hospital, Ruian People’s Hospital, Yongjia People’s Hospital, and Pingyang People’s Hospital) participated in this multicenter, prospective study. Patients who were admitted to the hospital within one week after the sudden onset of stroke between October 1 and December 31, 2018, were included in the data pool. Only patients with complete data on FBG and HbA1c upon admission were enrolled in this study. Stroke, including ischemic stroke and hemorrhagic stroke, was diagnosed according to the World Health Organization criteria [24] and confirmed by brain computed tomography or magnetic resonance imaging.

### Data collection

We collected data on patient demographics, medical history, neurological functional assessment, risk of malnutrition assessment and medical treatment during hospitalization. The baseline data were collected by the same professionally trained investigator within 48 h after admission. Whether the patient had a history of atrial fibrillation, coronary heart disease or stroke was determined on the basis of statements from the patient or their relatives and confirmed by a review of the patient’s past medical history. Hypertension and diabetes were assessed according to the 2010 Chinese guidelines for the management of hypertension [25] and the 2018 American Diabetes Association Standards of Medical Care in Diabetes [26], respectively. Hyperlipemia was diagnosed when one of the following criteria was met: total cholesterol level ≥ 6.2 mmol/L, low-density lipoprotein cholesterol level ≥ 4.1 mmol/L, triglyceride level ≥ 2.3 mmol/L or high-density lipoprotein cholesterol level < 1.0 mmol/L. The severity of stroke was assessed with the National Institutes of Health Stroke Scale (NIHSS) [27]. The Barthel index was used to assess the patient’s level of independence in activities of daily living (ADL) [28]. According to the Nutritional Risk Screening 2002, which considers impaired nutritional status, the severity of the disease and age, a total score of ≥ 3 points indicates that the patient is at risk of malnutrition [29]. Nutrition support was defined as enteral nutrition or intravenous nutrition support during hospitalization.

### Assessment of stress hyperglycemia

Fasting venous blood samples were drawn within 48 h after admission during the morning hours after an overnight fast to measure FBG and HbA1c. All measurements were performed by laboratory personnel blinded to the study samples, study group assignments, and outcomes.

Stress hyperglycemia was estimated with the FBG/HbA1c ratio. We used the following formula to calculate the FBG/HbA1c ratio: FBG/HbA1c ratio = FBG (mmol/L)/HbA1c (%). According to the quartiles of the FBG/HbA1c ratio, the patients were further categorized into four equal groups (Q1–Q4, Q1 ≤ 0.81, 0.82 ≤ Q2 < 0.91, 0.92 ≤ Q3 < 1.06, Q4 ≥ 1.07). The FBG/HbA1c ratio was used to quantify the extent of acute elevation of plasma glucose compared with the background plasma glucose levels.

### Outcome assessment

The outcomes were assessed at three time points after stroke: within 48 h after discharge (infectious complications, National Institutes of Health Stroke Scale, NIHSS score and dysfunction), at three months after discharge (all-cause death and functional outcomes) and at 12 months after discharge (all-cause death and functional outcomes). The primary outcome was all-cause mortality, and secondary outcomes were poor prognosis defined as infectious complications, a NIHSS score ≥ 6, a Barthel Index score ≤ 60, or a modified Rankin Scale (mRS) score of 3–6. Infectious complications were defined as pneumonia, UTI, gastrointestinal infection, sepsis, infective fever, and other infections during hospitalization. Dysfunction was defined as a Barthel Index value ≤ 60. Functional outcomes were measured with the mRS score. Poor functional outcomes were defined as a mRS score of 3–6. We followed up with the patients by telephone at the end of 3 and 12 months after discharge. Patients who could not be contacted by telephone after discharge were considered to be lost to follow-up.

### Statistical analysis

Categorical variables are presented as frequencies and percentages, while continuous variables are presented as the mean with standard deviation (SD) or median with interquartile range (IQR) when data were not normally distributed. Outliers were detected using residual examination**.** Categorical variables were analyzed using the chi-square test or Fisher’s exact test. Normally distributed continuous variables were analyzed using ANOVA. Continuous variables that were not normally distributed were analyzed with the Mann–Whitney U test and Kruskal–Wallis test.

The relationship between the quartiles of the FBG/HbA1c ratio and outcomes after stroke were evaluated by multivariable logistic regression models with the lowest quartile as the reference. Collinearity was checked before establishing each regression models. The tolerance ≥ 0.1 and the variance inflation factor < 10 were considered to be the absence of collinearity. The ratio of outcome events per independent variable were ≥ 10. For the regression analyses with infectious complications as outcomes, we adjusted for the following factors: sex, age, NIHSS score, white blood cell count (WBC), type of stroke, diabetes, previous stroke, alcohol abuse, risk of malnutrition, length of hospital stay, and nutrition support. For the regression analyses with other in-hospital outcomes, we adjusted for sex, age, NIHSS score, type of stroke, atrial fibrillation, hypertension, diabetes, coronary heart disease, hyperlipemia, previous stroke, history of smoking, and history of alcohol consumption. For the regression analyses with mortality and functional outcomes at 3 and 12 months after discharge, we adjusted for sex, age, NIHSS score, type of stroke, atrial fibrillation, hypertension, diabetes, coronary heart disease, hyperlipemia, previous stroke, history of smoking, history of alcohol consumption, and infectious complications during hospitalization. We performed a sensitivity analysis restricted to patients without a history of diabetes, as previous studies have suggested that this group of patients has a worse outcome when presenting with hyperglycemia [30].

Infection during hospitalization was significantly associated with the short-term risk of recurrent stroke [31]. To assess infectious complications as an intermediate in the pathway from stress hyperglycemia to mid- and long-term all-cause death, we also adjusted for infectious complications (both as an individual variable and as an interaction variable with stress hyperglycemia quartiles) in the multivariate regression models with all-cause mortality at 3 and 12 months.

To investigate the association between stress hyperglycemia and all-cause mortality at 12 months after stroke in relation to each variable, we performed subgroup analyses of the following adjusted variables: sex, age (< 75 or ≥ 75), NIHSS score (< 6 or ≥ 6), diabetes, hypertension, infectious complications, and stroke type (ischemic or hemorrhagic).

The predictive ability of HbA1c, FBG and the FBG/HbA1c ratio with primary outcomes was assessed using the receiver–operating characteristics (ROC) curve analysis.

Data were analyzed by SPSS 22.0 software (SPSS, Inc., Chicago, IL) and MedCalc (MedCalc Inc, Ostend, Belgium), and a 2-sided *P* value < 0.05 was considered to indicate statistical significance.

## Results

**Fig. 1:**
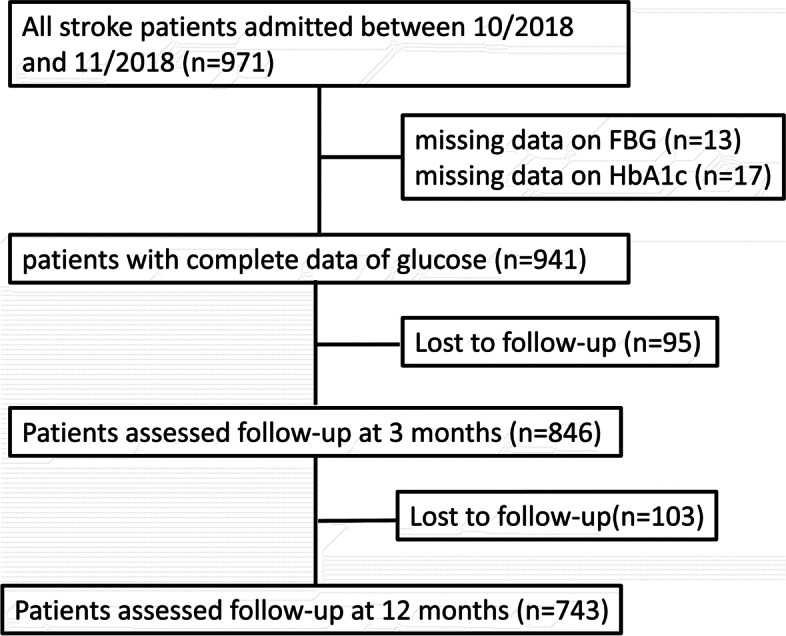
Flowchart of study participants

Among 971 stroke patients, 13 missed the data of FBG, 17 missed the data of HbA1c, 846 were successfully followed up with 3 months after discharge, and 743 of these patients remained in contact at 12 months (Fig. 1). Therefore, we performed most analyses with 846 patients, and the 12-month analysis was restricted to 743 patients. Of the 846 stroke patients, 522 (61.7%) were male, and the mean age was 65.7 ± 12.6 years. The mean and median FBG/HbA1c ratios were 0.98 (SD, 0.34) and 0.91 (IQR, 0.81–1.06), respectively. The median of admission NIHSS score was 3 (IQR, 1–7). (Table 1) shows the baseline characteristics of the patients by quartiles of the FBG/HbA1c ratio.

**Table 1:** Baseline characteristics

	FBG/HbA1c				*p* Value
	Q1 (*n* = 214)	Q2 (*n* = 212)	Q3 (*n* = 210)	Q4 (*n* = 210)	
Patient characteristics					
Female sex, n (%)	61 (28.5)	95 (44.8)	79 (37.6)	89 (42.4)	0.003
Age, mean (SD), years	66.3 (11.8)	66.1 (13.1)	64.8 (12.8)	65.5 (12.6)	0.624
NIHSS, median (IQR)	3 (1–5)	2 (1–5)	3 (1–6.5)	4 (1–9.5)	< 0.001
Barthel Index, median (IQR)	75 (45–95)	75 (45–95)	60 (30–92.5)	45 (20–75)	< 0.001
Risk of malnutrition, n (%)	91 (42.7)	87 (41.0)	85 (40.5)	99 (47.5)	0.503
Nutrition support, n (%)	11 (5.1)	11 (5.2)	26 (12.4)	49 (23.3)	< 0.001
Alcohol abuse, n (%)	34 (15.9)	25 (11.8)	30 (14.3)	24 (11.4)	0.482
Length of hospital stay, median (IQR), days	10 (8–13)	9 (7–13)	10 (7–14)	12 (8–17)	< 0.001
Type of stroke, n (%)					< 0.001
Ischemic	162 (75.7)	155 (73.1)	134 (63.8)	113 (53.8)	
Hemorrhagic	52 (24.3)	57 (26.9)	76 (36.2)	97 (46.2)	
Cardiovascular risk factors, n (%)					
Atrial fibrillation	18 (8.4)	12 (5.7)	11 (5.2)	21 (10.0)	0.187
Hypertension	151 (70.6)	143 (67.5)	159 (75.7)	160 (76.2)	0.131
Diabetes mellitus	65 (30.4)	42 (19.8)	65 (31.0)	97 (46.2)	< 0.001
Coronary heart disease	14 (6.5)	6 (2.8)	8 (3.8)	9 (4.3)	0.286
Hyperlipemia	89 (41.6)	111 (52.4)	105 (50.0)	109 (51.9)	0.092
Previous stroke	58 (27.1)	39 (18.4)	38 (18.1)	33 (15.7)	0.018
History of smoking	75 (35.0)	57 (26.9)	61 (29.0)	55 (26.2)	0.172
History of drinking	72 (33.6)	55 (25.9)	61 (29.0)	56 (26.7)	0.289
Laboratory indicators					
FBG, mean (SD), mmol/L	4.88 (1.1)	5.34 (1.0)	6.28 (1.3)	9.37 (4.0)	< 0.001
HbA1C, mean (SD), %	6.75 (1.7)	6.18 (1.1)	6.41 (1.3)	6.99 (2.0)	< 0.001
FBG/HbA1C, mean (SD)	0.73 (0.09)	0.86 (0.02)	0.98 (0.04)	1.34 (0.49)	< 0.001
WBC, mean (SD), 109/L	6.95 (1.91)	6.70 (1.99)	7.40 (2.32)	8.18 (3.12)	< 0.001

Quartiles of FBG/HbA1c ratio, Q1 ≤ 0.81, 0.82 ≤ Q2 < 0.91, 0.92 ≤ Q3 < 1.06, Q4 ≥ 1.07

*FBG* Fasting blood glucose, *HbA1c* Glycated hemoglobin, *NIHSS* The National Institutes of Health Stroke Scale, *WBC* White blood cell count

The characteristics of patients included and excluded were showed in Additional file 1.

### Stress hyperglycemia and primary outcomes

**Fig. 2:**
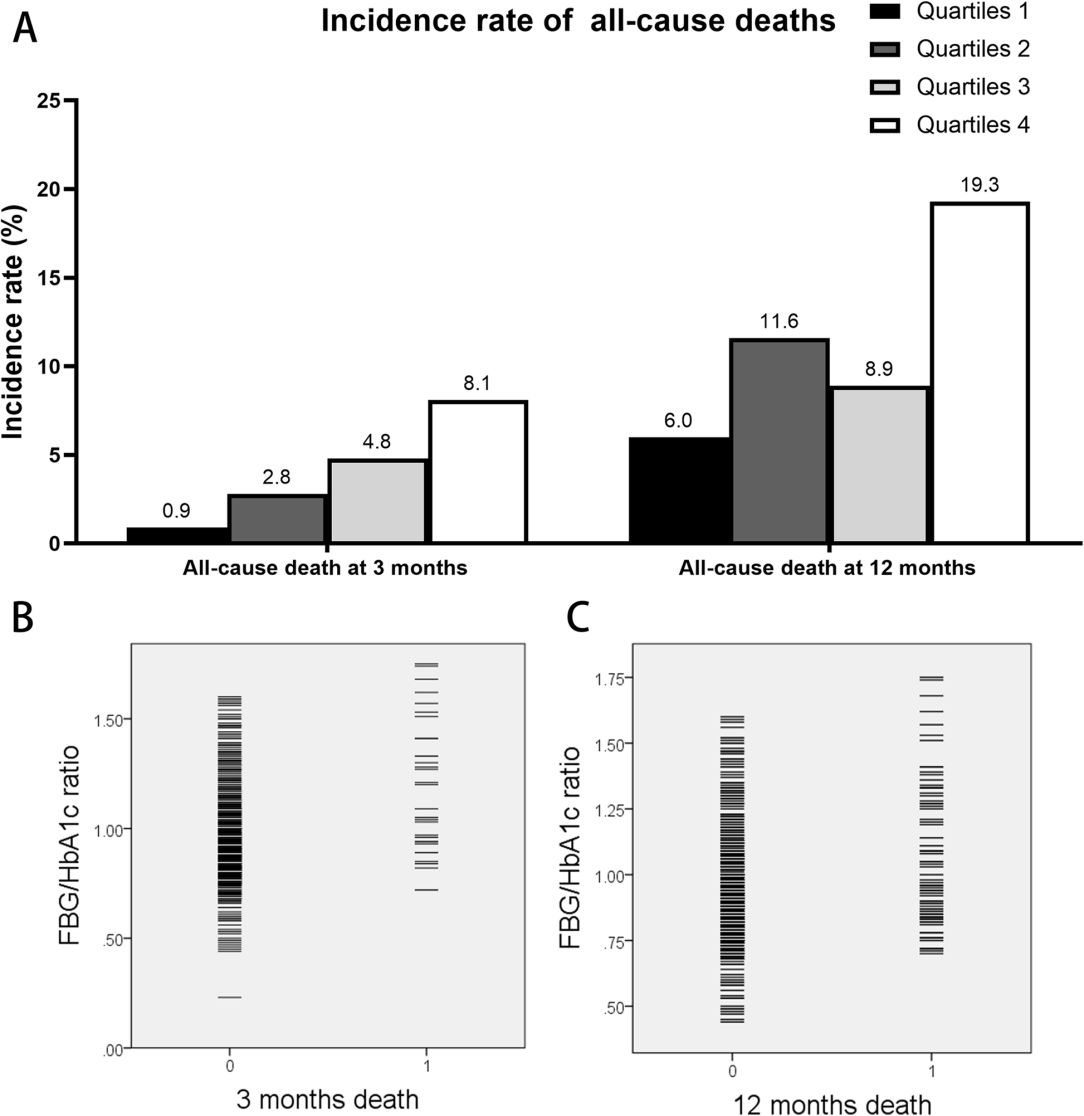
The association between FBG/HbA1c ratio and all-cause deaths. **A** The incidence rate of 3- and 12-month all-cause mortality after stroke across quartiles of the FBG/HbA1c ratio. **B** The scatterplot of the relationship between the FBG/HbA1c ratio and 3-month all-cause deaths. *0* survival, *1* death. **C** The scatterplot of the relationship between the FBG/HbA1c ratio and 12-month all-cause deaths. *0* survival, *1* death

(Table 2) shows the 3- and 12-month all-cause mortality after stroke across quartiles of the FBG/HbA1c ratio. (Figure 2) shows the histograms and scatterplots of association between FBG/HbA1c ratio and all-cause deaths. There were 35 (4.1%) all-cause deaths at 3 months and 85 (11.4%) at 12 months. After adjustment for sex, age, NIHSS, atrial fibrillation, hypertension, diabetes, coronary heart disease, hyperlipemia, previous smoking, history of smoking, history of drinking, and infectious complications, multivariable regression showed that patients in the highest quartile of the FBG/HbA1c ratio had an elevated risk of all-cause death at 3 months (adjusted OR: 5.16, 95% CI: 1.03–25.74) and at 12 months (adjusted OR: 2.59, 95% CI: 1.14–5.89).

**Table 2:** Logistic regression of all-cause death according to FBG/HbA1c quartiles

Outcomes	FBG/HbA1c	n	Events, n (%)	Crude OR (95% CI)	*p* Value	Adjusted OR (95% CI)^a^	*p* Value
Primary Outcomes							
All-cause death at 3 months	Q1 (≤ 0.81)	214	2 (0.9)	Ref		Ref	
	Q2 (0.82–0.91)	212	6 (2.8)	3.09 (0.62–15.48)	0.170	2.86 (0.54–15.24)	0.218
	Q3 (0.92–1.06)	210	10 (4.8)	5.30 (1.15–24.49)	0.033	3.95 (0.81–19.35)	0.090
	Q4 (≥ 1.07)	210	17 (8.1)	9.34 (2.13–40.94)	0.003	5.16 (1.03–25.74)	0.045
All-cause death at 12 months	Q1 (≤ 0.81)	183	11 (6.0)	Ref		Ref	
	Q2 (0.82–0.91)	189	22 (11.6)	2.06 (0.97–4.38)	0.060	2.47 (1.09–5.59)	0.030
	Q3 (0.92–1.06)	190	17 (8.9)	1.54 (0.70–3.38)	0.285	1.31 (0.56–3.07)	0.534
	Q4 (≥ 1.07)	181	35 (19.3)	3.78 (1.84–7.64)	< 0.001	2.59 (1.14–5.89)	0.024

Quartiles of FBG/HbA1c ratio, Q1 ≤ 0.81, 0.82 ≤ Q2 < 0.91, 0.92 ≤ Q3 < 1.06, Q4 ≥ 1.07

*FBG* Fasting blood glucose, *HbA1c* Glycated hemoglobin

^a^Logistic regression is adjusted for sex, age, NIHSS, type of stroke, atrial fibrillation, hypertension, diabetes, coronary heart disease, hyperlipemia, previous stroke, history of smoking, history of drinking, and infectious complications

### Stress hyperglycemia and secondary outcomes

(Table 3) shows the outcomes during hospitalization across quartiles of the FBG/HbA1c ratio in the study patients. There were 140 (16.5%) infectious complications, including 102 (12.1%) instances of pneumonia and 22 (2.6%) UTIs. A total of 165 (19.5%) patients had NIHSS scores ≥ 6, and 337 (39.8%) had dysfunction (Barthel index value ≤ 60) after discharge. Compared with patients in the lowest quartile, patients in the highest quartile of the FBG/HbA1c ratio had higher NIHSS scores (*P* < 0.001). Compared with patients in the lowest quartile, patients in the highest quartile of the FBG/HbA1c ratio had a higher risk of infectious complications (adjusted OR 2.37, 95% CI 1.27–4.43) and dysfunction (adjusted OR 1.79, 95% CI 1.06–3.01) after adjusting for covariates. There was also a trend that patients in the highest quartile of the FBG/HbA1c ratio had a higher risk of pneumonia (adjusted OR 1.44, 95% CI 0.73–2.82) and UTI (adjusted OR 2.32, 95% CI 0.53–10.13) and a higher prevalence of NIHSS score ≥ 6 (adjusted OR 1.63, 95% CI 0.83–3.22) than patients in the lowest quartile.

**Table 3:** Logistic regression of outcomes during hospitalization according to FBG/HbA1c quartiles

Outcomes	FBG/HbA1c	n	Events, n (%)	Crude OR (95% CI)	*p* Value	Adjusted OR (95% CI)	*p* Value
Secondary outcomes: during hospitalization							
Infectious complications^a^	Q1 (≤ 0.81)	214	21 (9.8)	Ref		Ref	
	Q2 (0.82–0.91)	212	23 (10.8)	1.12 (0.60–2.09)	0.725	1.13 (0.56–2.24)	0.738
	Q3 (0.92–1.06)	210	29 (13.8)	1.47 (0.81–2.68)	0.204	1.14 (0.58–2.23)	0.706
	Q4 (≥ 1.07)	210	67 (31.9)	4.31 (2.52–7.36)	< 0.001	2.37 (1.27–4.43)	0.007
Pneumonia^a^	Q1 (≤ 0.81)	214	19 (8.9)	Ref		Ref	
	Q2 (0.82–0.91)	212	14 (6.6)	0.73 (0.35–1.49)	0.381	0.70 (0.30–1.47)	0.669
	Q3 (0.92–1.06)	210	21 (10.0)	1.14 (0.59–2.29)	0.693	0.82 (0.39–1.70)	0.818
	Q4 (≥ 1.07)	210	48 (22.9)	3.04 (1.72–5.38)	< 0.001	1.44 (0.73–2.82)	0.573
Urinary tract infection^a^	Q1 (≤ 0.81)	214	3 (1.4)	Ref		Ref	
	Q2 (0.82–0.91)	212	6 (2.8)	2.05 (0.51–8.30)	0.315	1.67 (0.39–7.14)	0.488
	Q3 (0.92–1.06)	210	5 (2.4)	1.72 (0.41–7.27)	0.464	1.71 (0.38–7.63)	0.458
	Q4 (≥ 1.07)	210	8 (3.8)	2.78 (0.73–10.65)	0.134	2.32 (0.53–10.13)	0.265
NIHSS ≥ 6 post discharge^b^	Q1 (≤ 0.81)	214	29 (14.1)	Ref		Ref	
	Q2 (0.82–0.91)	212	30 (14.7)	1.05 (0.61–1.83)	0.856	1.22 (0.60–2.49)	0.585
	Q3 (0.92–1.06)	210	39 (19.4)	1.47 (0.87–2.49)	0.151	0.81 (0.40–1.62)	0.546
	Q4 (≥ 1.07)	210	67 (34.0)	3.15 (1.93–5.14)	< 0.001	1.63 (0.83–3.22)	0.156
Dysfunction post discharge (Bathel ≤ 60)^b^	Q1 (≤ 0.81)	214	81 (31.9)	Ref		Ref	
	Q2 (0.82–0.91)	212	88 (41.5)	1.11 (0.74–1.66)	0.630	1.06 (0.64–1.79)	0.818
	Q3 (0.92–1.06)	210	114 (51.3)	1.56 (1.04–2.32)	0.030	1.22 (0.74–20.3)	0.442
	Q4 (≥ 1.07)	210	139 (66.2)	2.72 (1.82–4.04)	< 0.001	1.79 (1.06–3.01)	0.029

Quartiles of FBG/HbA1c ratio, Q1 ≤ 0.81, 0.82 ≤ Q2 < 0.91, 0.92 ≤ Q3 < 1.06, Q4 ≥ 1.07

*FBG* Fasting blood glucose, *HbA1c* Glycated hemoglobin, *NIHSS* The National Institutes of Health Stroke Scale

^a^Adjusted for sex, age, NIHSS, WBC, type of stroke, diabetes, previous stroke, alcohol abuse, risk of malnutrition, length of hospital stay, and nutrition support

^b^Adjusted for sex, age, NIHSS, type of stroke, atrial fibrillation, hypertension, diabetes, coronary heart disease, hyperlipemia, previous stroke, history of smoking and history of drinking

(Table 4) shows the 3- and 12-month poor functional outcomes after stroke across quartiles of the FBG/HbA1c ratio. There were 204 (24.1%) poor outcomes at 3 months and 241 (32.9%) at 12 months. There was a trend that patients in the highest quartile of the FBG/HbA1c ratio had a higher risk of poor functional outcomes at 3 months (adjusted OR 1.33, 95% CI 0.75–2.36) and 12 months (adjusted OR 1.68, 95% CI 0.97–2.90).

**Table 4:** Logistic regression of poor functional outcomes according to FBG/HbA1c quartiles

Outcomes	FBG/HbA1c	n	Events, n (%)	Crude OR (95% CI)	*p* Value	Adjusted OR (95% CI)^a^	*p* Value
Secondary Outcomes: after discharge							
Poor functional outcomes (mRS of 3–6) at 3 months	Q1 (≤ 0.81)	214	32 (16.4)	Ref		Ref	
	Q2 (0.82–0.91)	212	46 (21.7)	1.42 (0.87–2.31)	0.161	1.50 (0.85–2.65)	0.159
	Q3 (0.92–1.06)	210	50 (23.8)	1.64 (1.02–2.65)	0.043	1.16 (0.66–2.05)	0.601
	Q4 (≥ 1.07)	210	72 (34.3)	2.67 (1.68–4.23)	< 0.001	1.33 (0.75–2.36)	0.341
Poor functional outcomes (mRS of 3–6) at 12 months	Q1 (≤ 0.81)	183	52 (28.4)	Ref		Ref	
	Q2 (0.82–0.91)	189	49 (25.9)	0.88 (0.56–1.39)	0.589	1.16 (0.68–1.99)	0.582
	Q3 (0.92–1.06)	190	57 (30.0)	1.08 (0.69–1.69)	0.737	0.94 (0.55–1.59)	0.803
	Q4 (≥ 1.07)	181	83 (45.9)	2.13 (1.38–3.29)	0.001	1.68 (0.97–2.90)	0.062

Quartiles of FBG/HbA1c ratio, Q1 ≤ 0.81, 0.82 ≤ Q2 < 0.91, 0.92 ≤ Q3 < 1.06, Q4 ≥ 1.07

*FBG* Fasting blood glucose, *HbA1c* Glycated hemoglobin

^a^Logistic regression is adjusted for sex, age, NIHSS, type of stroke, atrial fibrillation, hypertension, diabetes, coronary heart disease, hyperlipemia, previous stroke, history of smoking, history of drinking, and infectious complications

The sensitivity analyses excluding patients with preexisting diabetes yielded similar results (Additional file 2).

### The role of infectious complications in the pathway from admission stress hyperglycemia to death

To assess the role of infectious complications in the pathway from stress hyperglycemia to mid- and long-term all-cause death, we also added infectious complications as an interaction variable with stress hyperglycemia quartiles in the logistic regression model. Multivariable regression showed that adding infectious complications (both as individual variables and as an interaction variable with quartiles of FBG/HbA1c ratio) to the models did not modify the relationships of the highest quartile of the FBG/HbA1c ratio with all-cause death at 3 months (adjusted OR 5.63, 95% CI 1.05–30.16) and at 12 months (adjusted OR 3.36, 95% CI 1.28–8.85). No statistical significance was found in the interaction of the FBG/HbA1c ratio with infectious complications in predicting all-cause mortality at 3 and 12 months (*P* = 0.516 and *P* = 0.369, respectively).

### Subgroup analysis

**Fig. 3:**
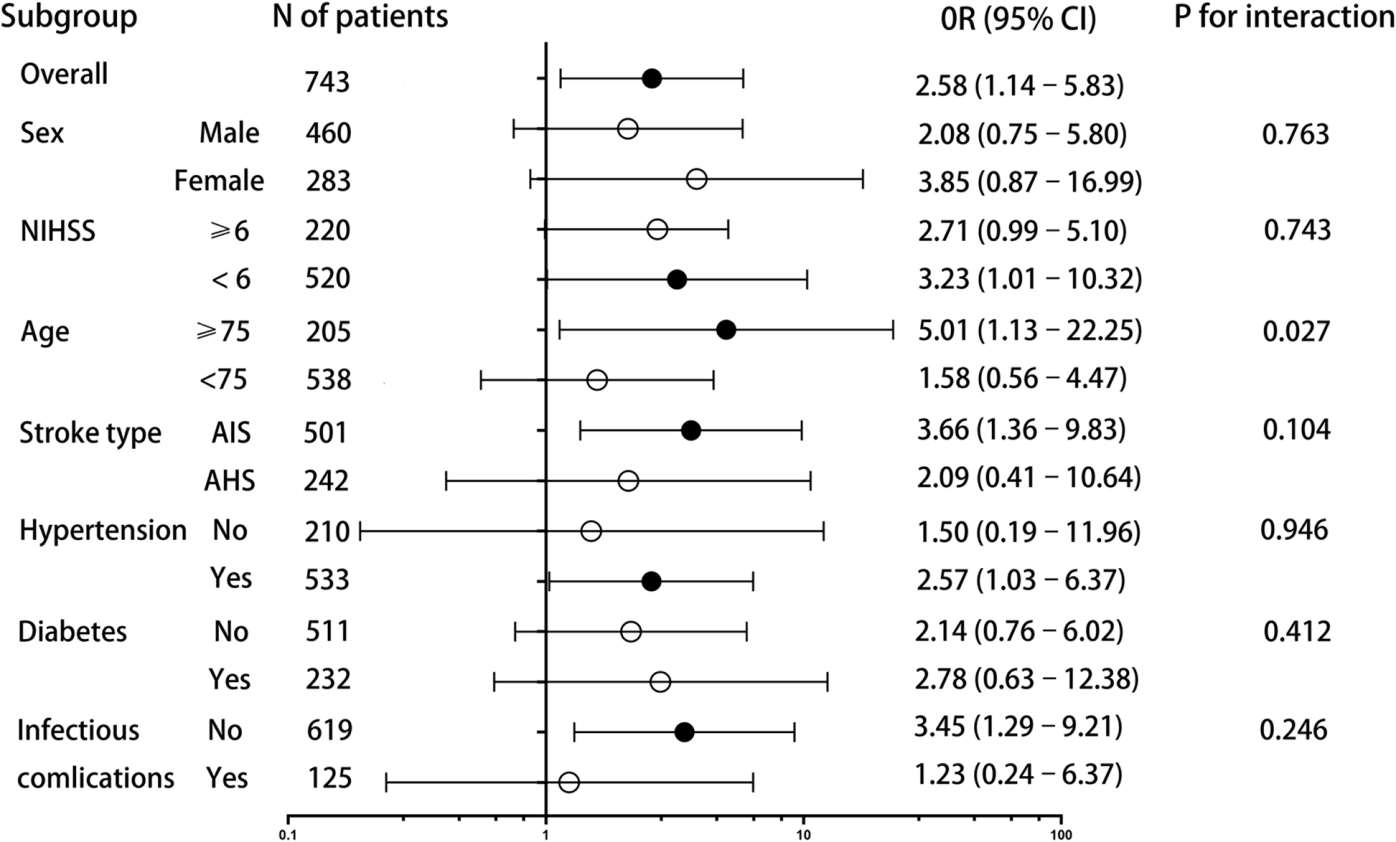
Logistic regression of 12-month all-cause death according to FBG/HbA1c quartiles in subgroup analysis

In the subgroup analysis of all-cause mortality at 12 months, the highest quartile of the FBG/HbA1c ratio was associated with or showed a trend toward an association with infectious complications in all subgroups (Fig. 3). Compared with patients in the lowest quartile, patients in the highest quartile of the FBG/HbA1c ratio had a higher risk of infectious complications in the subgroups of NIHSS score < 6 (adjusted OR 3.23, 95% CI 1.01–10.32), age ≥ 75 (adjusted OR 5.01, 95% CI 1.13–22.25), AIS subjects (adjusted OR 3.66, 95% CI 1.36–9.83), hypertensive subjects (adjusted OR 2.57, 95% CI 1.03–6.37), and noninfectious subjects (adjusted OR 3.45, 95% CI 1.29–9.21).

### Comparison of the predictive ability of HbA1c, FBG and the FBG/HbA1c ratio with primary outcomes

At ROC analysis (Additional file 3, Fig. 4), based on Area Under the Curve, the FBG/HbA1c ratio was shown to be better than FBG in predicting 3 months mortality (0.704 vs. 0.616, *P* < 0.001). The predictive ability of the FBG/HbA1c ratio showed to be better than FBG (0.619 vs. 0.579, *P* = 0.049) and HbA1c (0.619 vs. 0.522, *P* = 0.050) in predicting 12 months mortality, respectively.

**Fig. 4:**
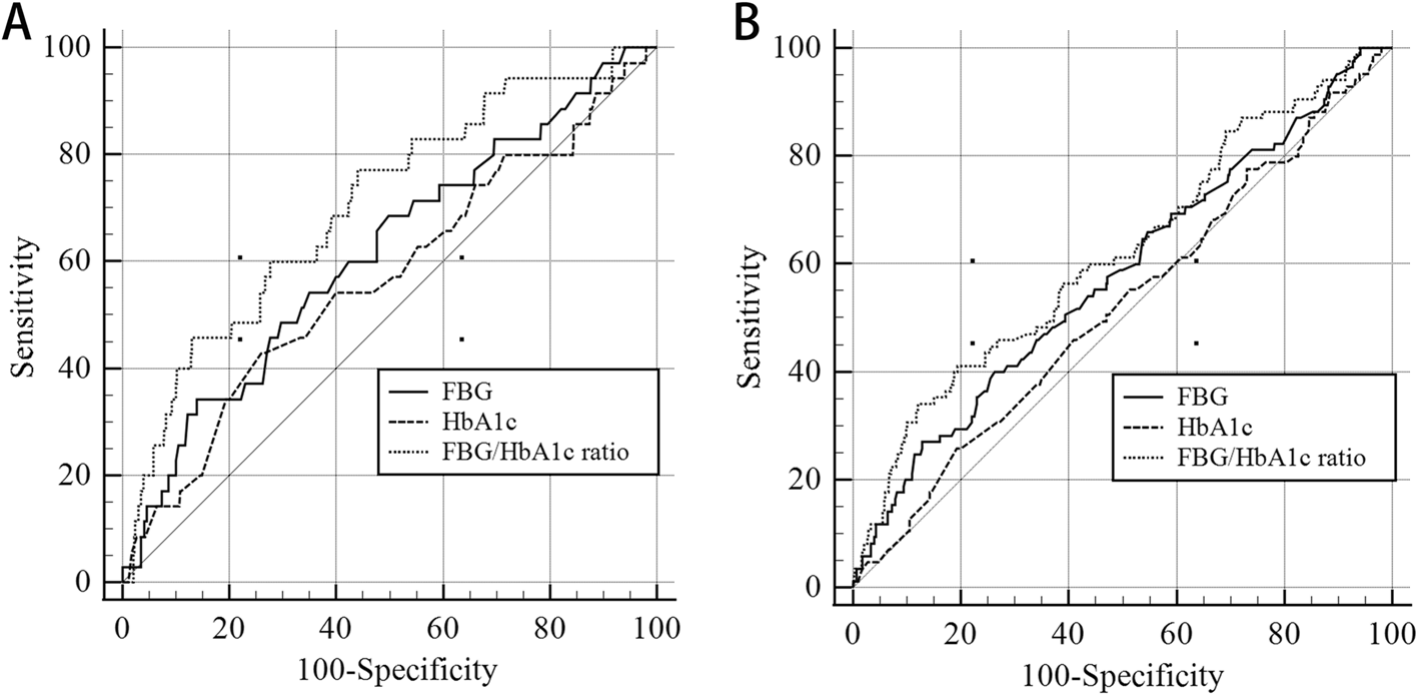
ROC curve analysis for comparing FBG, HbA1c, and FBG/HbA1c ratio with 3- and 12- month all-cause mortality. **A** FBG, HbA1c, and FBG/HbA1c ratio with 3-month all-cause mortality. **B** FBG, HbA1c, and FBG/HbA1c ratio with 12-month all-cause mortality

## Discussion

In this multicenter, prospective study, we explored the association between stress hyperglycemia and all-cause mortality and poor prognosis in patients with acute stroke and found that the glucose/HbA1c ratio may independently predict mid- and long-term all-cause mortality (adjusted OR: 2.59, 95% CI: 1.14–5.89), infectious complications (adjusted OR 2.37, 95% CI 1.27–4.43), and dysfunction (adjusted OR 1.79, 95% CI 1.06–3.01) in acute stroke patients. There are several types of markers of stress hyperglycemia. Absolute increasing in admission glucose level was found to be associated with in-hospital mortality after acute stroke in earlier studies [30, 32]. Recent studies have shown that relative hyperglycemia, defined as the glucose to HbA1c ratio [12–14] or as admission glucose divided by the estimated average glucose level derived from HbA1c [15], could work as an economical and practical indicator to precisely detect and quantify stress hyperglycemia. The present study also revealed that relative glucose levels were much better than absolute glucose indicators in predicting 3 months and 12 months mortality (Additional file 3 and Fig. 4). Besides glucose/HbA1c ratio, glucose/glycated albumin ratio was shown to be associated with an increased risk of stroke in patients with a minor ischemic stroke or transient ischemic attack [6]. The advantage of HbA1c is that it has been proposed to be a valuable diagnostic tool for monitoring long-term glycemic control for about 2 to 4 months [10] while glycated albumin level reflects mean glycemia level over 2 to 4 weeks [33]. Besides, HbA1c appears to be rather affordable and routine compared with glycated albumin.

Our current study showed that patients in the highest quartile of the FBG/HbA1c ratio had an increased risk of all-cause death at 3 months (adjusted OR: 5.16, 95% CI: 1.03–25.74) and at 12 months (adjusted OR: 2.59, 95% CI: 1.14–5.89) after adjusting for confounders, including demographic variables, stroke severity and cardiovascular risk factors. These results demonstrated the independent role of stress hyperglycemia in predicting mid- and long-term all-cause death in acute stroke patients.

One of our secondary outcomes was poor functional outcomes defined as a mRS score of ≥ 3. Our results revealed that there was a trend that patients in the highest quartile of the FBG/HbA1c ratio had a higher risk of poor functional outcomes at 3 months (adjusted OR 1.33, 95% CI 0.75–2.36) and 12 months (adjusted OR 1.68, 95% CI 0.97–2.90). Since poor functional outcomes measured by the mRS might be most relevant to clinicians and patients considering further rehabilitation [34], these results suggested that stress hyperglycemia measured by the FBG/HbA1c ratio might facilitate the identification of patients with acute stroke who require early intervention to promote functional recovery.

Since a history of diabetes has been shown to be an important potential covariate in the relationship between stress hyperglycemia, complications and poor prognosis [35], sensitivity analyses excluding patients with preexisting diabetes were performed and yielded results similar to those of the overall analysis. Among nondiabetic patients, compared with patients in the lowest quartile, patients in the highest quartile of the FBG/HbA1c ratio had a higher risk of poor functional outcomes at 3 months and 12 months, which was consistent with a previous study [35]. These results suggested that the relationship between stress hyperglycemia and long-term adverse outcomes exists regardless of the presence or absence of a history of diabetes.

Another important secondary outcome was infectious complications of hospitalization. Infection is regarded as the most frequent complication of stroke, affecting almost 30% of patients [17], and has been confirmed to be an independent risk factor for a high risk of stroke recurrence [31]. Special attention should be given to the relationship between stress hyperglycemia and the risk of infectious complications after stroke. We found that stress hyperglycemia was associated with an increased risk of infectious complications (adjusted OR 2.37, 95% CI 1.27–4.43), which was consistent with a previous study [36] and added evidence on the relationship between disorders of glucose metabolism and complications in stroke patients [37, 38]. The association between hyperglycemia and infections after stroke may be explained by the fact that high blood glucose levels aggravate the already immunocompromised state of stroke patients [39, 40].

Despite the clear associations we found between stress hyperglycemia and infectious complications, we did not find evidence that the association between admission hyperglycemia and mid- and long-term all-cause death is explained or mediated by the occurrence of infectious complications (*P* = 0.516 and *P* = 0.369, respectively). This might be explained by the relatively low incidence rate of infectious complications in our population (16.5% vs. 30% in a systematic review [17]). Overall, further designed studies should be performed to explore the role of infections in hyperglycemia and worse clinical outcomes.

In the subgroup analysis of 12-month all-cause death, compared with patients in the lowest quartile, patients in the highest quartile of the FBG/HbA1c ratio had a higher risk of all-cause death in the subgroups of NIHSS score < 6, age ≥ 75, hypertensive subjects, and noninfectious subjects, which demonstrated the importance of the enhanced detection of stress hyperglycemia in these populations. Since the subgroup analyses were conducted post hoc, these associations should be explored in future research.

There are several explanations for the association between stress hyperglycemia and poor prognoses of stroke. First, stress hyperglycemia may contribute to adverse outcomes in stroke patients through mechanisms such as induction of more endothelial apoptosis and greater endothelial dysfunction and oxidative stress responses [1]. Second, hyperglycemia may have direct neurotoxicity on the ischemic penumbra and cause more neurons to be injured^3^, which may facilitate the conversion of hypoperfused at-risk tissue into infarction and adversely affect stroke prognosis [41]. Finally, acute hyperglycemia increases brain lactate production, which may accelerate irreversible injury by enhancing glutamate release and altering intracellular calcium regulation [42, 43], thus resulting in poorer stroke outcomes.

Although clear associations of stress hyperglycemia with all-cause death were found in the present study, a recent observational study [9] and a clinical trial [44] have come to the opposite conclusion. An observational study showed that stress hyperglycemia measured by absolute fasting serum glucose levels was not directly associated with in-hospital mortality but was only a marker of stroke severity [9]. The conflicting data presented above, which highlight a controversial role for stress hyperglycemia on outcomes following stroke, may result from the discrepancy in the measurement time points (at the second day after admission) and the index for hyperglycemia (absolute but not relative increases in fasting glucose were assessed in this study). Moreover, the present study showed that after adjusting for the NIHSS score as one of the confounders in the multivariable model, the associations remained significant between stress hyperglycemia and an increased risk of all-cause death at 3 months and 12 months after stroke, respectively. The recent randomized Stroke Hyperglycemia Insulin Network Effort trial, which compared intensive glucose lowering with standard treatment in patients with stroke, found no difference in the death and favorable mRS score at 3 months [44]. However, treatment with intravenous tissue plasminogen activator therapy in 63% of the patients may suggest a selection bias for patients in this study. Thus, further well-designed randomized clinical trials with larger samples should be performed to evaluate whether improving glycemic control in stroke patients could help to achieve better outcomes.

There are several strengths in our present study. The data presented in this study likely reflect daily clinical practice, as they come from consecutive patients at 5 major hospitals in the Wenzhou region in China who were treated for stroke without the exclusion of hemorrhagic stroke patients, unlike most previous studies [6, 7, 36]. In addition, the present study prospectively designed abundant prognostic indicators. The primary outcome was all-cause mortality, and the secondary outcomes were poor prognosis defined as infectious complications, NIHSS score ≥ 6, Barthel Index score ≤ 60, and mRS score ≥ 3. The inclusion of both short- and long-term follow-up information strengthened the reliability of our results. There are still some limitations. First, 95 patients were lost to follow-up at 3 months, and 103 were lost at 12 months after discharge, which may lead to selection bias. Second, recall bias and underdiagnosis might result in the misclassification of confounding factors. Besides, there were no data available on the time elapsed after the use of glucose-lowering medication or last meal, and on how well glucose levels was controlled during the follow-up period as well as the medicines for prevention of cardiovascular event recurrence. Therefore, we were unable to assess the dynamics of hyperglycemia after stroke, although studies have suggested that persistent hyperglycemia is associated with poor functional outcomes [45]. Moreover, the present study lacks information on baseline markers of infection relating to the analysis of in-hospital infections except for WBC count.

In conclusion, stress hyperglycemia on admission measured by the FBG/HbA1c ratio was associated with an increased risk of all-cause death after stroke. Our present study suggests that the glucose/HbA1c ratio may help identify patients at increased risk of adverse outcomes, including infectious complications, dysfunction, and all-cause death. Further prospective studies or randomized trials with large sample sizes are warranted to evaluate whether improving glycemic control in stroke patients could improve outcomes.

## Supplementary Information

**Additional file 1.** Baseline characteristics of patients included and excluded.

**Additional file 2.** Logistic regression of outcomes according to FBG/HbA1c quartiles in nondiabetic patients.

**Additional file 3.** Comparison of the predictive ability of HbA1c, FBG and the FBG/HbA1c ratio with mortality.

## Data Availability

The datasets used and analysed during the current study are available from the corresponding author on reasonable request.

## Abbreviations

FBG: Fasting blood glucose
HbA1c: Glycated hemoglobin
UTI: Urinary tract infection
NIHSS: The National Institutes of Health Stroke Scale
ADL: Activities of daily living
mRS: The modified Rankin Scale
