# Supplementary material for: Fasting blood glucose-to-glycated hemoglobin ratio and all-cause mortality among Chinese in-hospital patients with acute stroke: a 12-month follow-up study
Source: BMC Geriatr. 2022 Jun 20;22:508. doi: 10.1186/s12877-022-03203-3 (PMC9210760; doi:10.1186/s12877-022-03203-3)
Supplement: Supplementary file 1 — Additional file 1. Baseline characteristics of patients included and excluded. [file 12877_2022_3203_MOESM1_ESM.pdf]

Additional file 1: Baseline characteristics of patients included and excluded.

	Included (n = 846)	Excluded (n = 125)	*p* Value
Patient characteristics			
Female sex, n (%)	324 (38.3)	41 (32.8)	0.236
Age, mean (SD), years	66.3 (11.8)	66.1 (13.1)	0.624
NIHSS, median (IQR)	3 (1–6)	2 (1–7.5)	0.227
Barthel Index, median (IQR)	60 (35–92.5)	50 (35–90)	0.097
Risk of malnutrition, n (%)	362 (42.8)	64 (51.2)	0.079
Nutrition support, n (%)	97 (11.5)	19 (15.2)	0.230
Length of hospital stay, median (IQR), days	10 (8–14)	10 (7–13)	0.357
Type of stroke, n (%)			0.556
Ischemic	564 (67.7)	80 (64.0)	
Hemorrhagic	282 (33.3)	45 (36.0)	
Cardiovascular risk factors, n (%)			
Atrial fibrillation	62 (7.3)	11 (8.8)	0.560
Hypertension	613 (72.5)	97 (77.6)	0.226
Diabetes mellitus	269 (31.8)	33 (26.4)	0.224
Coronary heart disease	37 (4.4)	5 (4.0)	0.848
Hyperlipemia	414 (48.9)	50 (40.0)	0.062
Previous stroke	168 (19.9)	25 (20.0)	0.970
History of smoking	248 (29.3)	45 (36.0)	0.129
History of drinking	244 (28.8)	46 (36.8)	0.070

Abbreviations: NIHSS, The National Institutes of Health Stroke Scale
