# Supplementary material for: Fasting blood glucose-to-glycated hemoglobin ratio and all-cause mortality among Chinese in-hospital patients with acute stroke: a 12-month follow-up study
Source: BMC Geriatr. 2022 Jun 20;22:508. doi: 10.1186/s12877-022-03203-3 (PMC9210760; doi:10.1186/s12877-022-03203-3)
Supplement: Supplementary file 2 — Additional file 2. Logistic regression of outcomes according to FBG/HbA1c quartiles in nondiabetic patients. [file 12877_2022_3203_MOESM2_ESM.pdf]

Additional file 2: Logistic regression of outcomes according to FBG/HbA1c quartiles in nondiabetic patients.

Outcomes	FBG/HbA1C	n	Events, n (%)	Crude OR (95% CI)	Adjusted OR (95% CI)
Infectious complications[a]	Q1 (≤0.81)	145	12 (8.1)	Ref.	Ref.
	Q2 (0.82–0.91)	170	20 (11.8)	1.52 (0.72–3.23)	1.40 (0.58–3.37)
	Q3 (0.92–1.06)	145	22 (15.2)	2.04 (0.97–4.30)	1.33 (0.55–3.20)
	Q4 (≥1.07)	113	40 (35.4)	6.26 (3.09–12.66)	2.93 (1.23–6.95)
At 3 months					
Poor functional outcomes (mRS of 3–6)[b]	Q1 (≤0.81)	145	22 (14.8)	Ref.	Ref.
	Q2 (0.82–0.91)	170	37 (21.8)	1.61 (0.90–2.87)	1.80 (0.91–3.56)
	Q3 (0.92–1.06)	145	43 (29.7)	2.43 (1.37–4.33)	1.58 (0.80–3.14)
	Q4 (≥1.07)	113	45 (39.8)	3.82 (2.12–6.88)	2.40 (1.15–5.00)
All-cause death [b]	Q1 (≤0.81)	145	2 (1.3)	Ref.	Ref.
	Q2 (0.82–0.91)	170	6 (3.5)	2.69 (0.53–13.53)	2.69 (0.50–14.41)
	Q3 (0.92–1.06)	145	9 (6.2)	4.86 (1.03–22.91)	3.28 (0.66–16.43)
	Q4 (≥1.07)	113	10 (8.8)	7.14 (1.53–33.25)	3.81 (0.73–19.84)
At 12 months					
Poor functional outcomes (mRS of 3–6) [b]	Q1 (≤0.81)	127	34 (26.8)	Ref.	Ref.
	Q2 (0.82–0.91)	151	39 (25.8)	0.95 (0.56–1.63)	1.28 (0.69–2.38)
	Q3 (0.92–1.06)	132	40 (30.3)	1.19 (0.69–2.04)	0.93 (0.49–1.77)
	Q4 (≥1.07)	101	49 (48.5)	2.58 (1.48–4.48)	2.19 (1.10–4.32)
All-cause death [b]	Q1 (≤0.81)	127	8 (6.3)	Ref.	Ref.
	Q2 (0.82–0.91)	151	20 (13.2)	2.27 (0.96–5.35)	3.03 (1.19–7.68)
	Q3 (0.92–1.06)	132	13 (9.8)	1.63 (0.65–4.06)	1.33 (0.49–3.62)
	Q4 (≥1.07)	101	19 (18.8)	3.45 (1.44–8.25)	2.55 (0.93–6.99)

Notes: Quartiles of FBG/HbA1c ratio, Q1≤0.81, 0.82≤Q2<0.91, 0.92≤Q3<1.1.06, Q4≥1.07. Abbreviations: FBG, fasting blood glucose; HbA1c, glycated hemoglobin; NIHSS, The National Institutes of Health Stroke Scale.

[a]Adjusted for sex, age, NIHSS, previous stroke, alcohol abuse, risk of malnutrition, length of hospital stay, and nutrition support

[b] Adjusted for sex, age, NIHSS, atrial fibrillation, hypertension, coronary heart disease, hyperlipemia, previous stroke, history of smoking, history of drinking and infectious complications.
