# Supplementary material for: Fasting blood glucose-to-glycated hemoglobin ratio and all-cause mortality among Chinese in-hospital patients with acute stroke: a 12-month follow-up study
Source: BMC Geriatr. 2022 Jun 20;22:508. doi: 10.1186/s12877-022-03203-3 (PMC9210760; doi:10.1186/s12877-022-03203-3)
Supplement: Supplementary file 3 — Additional file 3. Comparison of the predictive ability of HbA1c, FBG and the FBG/HbA1c ratio with mortality. [file 12877_2022_3203_MOESM3_ESM.pdf]

Additional file 3: Comparison of the predictive ability of HbA1c, FBG and the FBG/HbA1c ratio with mortality

	ROC Curve Analysis	
	AUC (95% CI)	*P*-Value vs. FBG/ HbA1c ratio
In predicting 3 months mortality		
FBG	0.616 (0.582–0.649)	<0.001
HbA1c	0.568 (0.534–0.602)	0.079
FBG/ HbA1c ratio	0.704 (0.672–0.735)	/
In predicting 12 months mortality		
FBG	0.579 (0.542–0.615)	0.049
HbA1c	0.522 (0.534–0.602)	0.050
FBG/ HbA1c ratio	0.619 (0.583–0.654)	/

Abbreviations: ROC, receiver-operating characteristic, AUC, area under curve by ROC curve analysis, FBG, fasting blood glucose; HbA1c, glycated hemoglobin
